# Occupational dermatoses associated with glove use among health professionals: an integrative review and analysis of surveillance data

**DOI:** 10.47626/1679-4435-2026-1632

**Published:** 2026-10-20

**Authors:** Natasha Cecília Silva dos Prazeres, Julianne Alves Machado

**Affiliations:** 1 Universidade Tiradentes, Medicina, Aracaju, SE, Brazil

**Keywords:** occupational dermatitis, health personnel, protective gloves, occupational medicine

## Abstract

Latex-associated occupational dermatitis is a relevant condition among health professionals, particularly because of the frequent, prolonged use of gloves. The aim of this study was to analyze the epidemiological information available on this condition in this population. An integrative literature review was conducted through searches of PubMed, VHL, SciELO, and Google Scholar, including studies published between 2020 and 2025. The final sample comprised 18 articles. In addition, national data available from DATASUS and information from international surveillance systems were consulted. The DATASUS search identified no records listing rubber as the etiological agent. Brazilian scientific literature on the subject was scarce, in contrast to the greater availability of international data, although these data also had limitations regarding the identification of the etiological agent. Research indicates the condition is multifactorial and associated with frequent and prolonged exposure, impaired skin barrier function, and a history of atopy. Manifestations were more frequent among women, possibly because of their greater representation in occupational groups characterized by high levels of exposure. The findings also suggested changes in the profile of dermatoses during the COVID-19 pandemic, inequalities in the implementation of regulatory measures, and possible underreporting in surveillance systems. In conclusion, specific epidemiological information on latex-associated occupational dermatitis remains limited, particularly in Brazil. Strengthening reporting mechanisms, accurately recording etiological agents, and conducting further studies are essential to determine the magnitude of this condition and guide prevention and occupational health surveillance strategies.

## INTRODUCTION

Skin is the body’s primary interface with external environment. Under normal conditions, it acts as a highly effective barrier against pathogens and potentially harmful chemical agents. However, a number of human activities, particularly those performed in occupational settings, may compromise its integrity. Frequent exposure to irritating chemicals and physical agents can impair the function of epidermal barrier and lead to inflammation, discomfort, and occupational dermatoses ^[1,2]^.

Occupational contact dermatitis is one of the most common conditions among these. It is an inflammatory skin disease triggered by exposure to irritants or allergens in the workplace. Clinical manifestations may range from acute lesions characterized by erythema, pruritus, and vesicles, to chronic conditions marked by dryness, skin thickening, fissures, and persistent inflammation. Hands and forearms are the most frequently affected areas because they are exposed to potentially harmful substances more often ^[3]^.

Health professionals are among the occupational groups most commonly affected because their work involves frequent contact with chemical agents, hygiene products, and personal protective equipment (PPE). The use of latex gloves is particularly relevant because health professionals constitute one of the main groups at risk of sensitization due to frequent contact with gloves and other materials used in patient care procedures ^[4-7]^.

Continuous exposure to latex proteins and the chemical additives used in glove manufacturing may trigger different clinical manifestations, posing a major occupational health and safety challenge. These manifestations may cause functional impairment, reduce quality of life, and adversely affect patient care ^[1,2,7]^. The main reactions associated with glove use include irritant contact dermatitis (ICD), allergic contact dermatitis (ACD), and immediate latex hypersensitivity, each of which has distinct pathophysiological characteristics.

The most common form, ICD is a nonimmune reaction resulting from direct, cumulative damage caused by chemical or physical agents. These agents may damage the skin barrier and induce inflammation, generally manifesting as subacute or chronic eczema ^[2,8]^.

In turn, ACD is a delayed type IV hypersensitivity reaction mediated by T lymphocytes in which haptens found primarily in the chemical additives used in rubber manufacturing, such as thiurams and dithiocarbamates, penetrate the skin and bind to proteins, forming antigenic complexes capable of inducing sensitization. Upon subsequent exposure, a delayed inflammatory response develops, characterized by eczema, pruritus, and vesiculation. A less common form is immediate type I hypersensitivity, which is mediated by IgE and may manifest as contact urticaria and angioedema; in severe cases, it can progress to anaphylaxis ^[5,8]^.

Research describes relevant aspects of this condition, including its risk factors, the groups most commonly affected, and prevention strategies. However, the available information remains fragmented and heterogeneous. Although international studies have assessed latex sensitization and latex-associated skin manifestations among health professionals, specific data from Brazil remain limited.

Official systems record occupational dermatoses in aggregate form, without systematically stratifying cases by causative agent. This makes it difficult to estimate the extent of latex-related dermatitis in Brazil ^[9]^. Additionally, there is a discrepancy between national and international data availability: while international studies and surveillance systems provide more extensive and detailed information, national databases such as those available through DATASUS contain few specific records ^[10]^.

The aim of current study was to analyze the availability of epidemiological information on latex-associated occupational dermatitis among health professionals through an integrative literature review and an examination of secondary data covering the period from 2020 to 2025. Specifically, it aimed to i) assess the availability of national data in DATASUS; ii) compare these data with information from international epidemiological surveillance systems; iii) characterize the most commonly affected groups, risk factors, and preventive measures; and iv) identify limitations related to data collection on this topic.

## METHODS

### STUDY DESIGN

This integrative review was supplemented by a documentary analysis of secondary data from publicly available national databases and international surveillance systems. It sought to identify and analyze recent publications on occupational dermatitis associated with glove use among health professionals to characterize the main variables related to its occurrence and determine the availability of records on this condition in the Brazilian Notifiable Diseases Information System (SINAN) and international surveillance systems.

### REVIEW QUESTION

The review question was: “What are the epidemiological characteristics of occupational contact dermatitis associated with glove use among health professionals in Brazil and other countries?” It was structured according to the PICo framework, in which P (population) corresponded to health professionals; I (phenomenon of interest), to occupational dermatitis associated with glove use, particularly latex exposure; and Co (context), to Brazilian and international settings, based on epidemiological studies published between 2020 and 2025.

### DATA SOURCES AND SEARCH STRATEGIES

Literature searches were conducted in PubMed, VHL, SciELO, and Google Scholar. The first three were the primary sources, whereas Google Scholar was used as a supplementary source to identify potentially relevant studies. Consensus platform was also used as an auxiliary tool during the initial identification of potentially relevant publications. Records identified using this tool were subsequently verified by the authors, classified according to their respective indexing databases, and assessed against predefined eligibility criteria.

Controlled vocabulary terms from MeSH and DeCS were combined using Boolean operators. The search strategy was based on the terms *healthcare workers*, *latex*, *occupational dermatitis*, and *epidemiology*, combined using the Boolean operator AND. Searches were adapted to the specific requirements of each source. We considered full-text articles published between 2020 and 2025 for which there were no initial language restrictions.

National data on records of occupational dermatitis from 2020 to 2025 were obtained from DATASUS ^[10]^. The TABNET platform was searched using the years of interest and the variable “etiologic agent.” Cases were selected using the L20-L30 of the International Statistical Classification of Diseases and Related Health Problems, 10th Revision (ICD-10) categories, which correspond to dermatitis and eczema. The search specifically investigated whether any records identified rubber as the etiologic agent.

International surveillance systems and institutional reports were also consulted to complement the analysis and contextualize the condition at the global level. The sources included the Health and Safety Executive (HSE) regulatory authority and The Health and Occupation Research Network (THOR).

### ELIGIBILITY CRITERIA

Original studies published between 2020 and 2025 were included if they involved health professionals, addressed dermatitis associated with glove use in an occupational setting, and reported data on prevalence, risk factors, or clinical manifestations. Studies were excluded if they did not include health professionals, investigate occupational dermatitis, address exposure associated with glove use, or report primary data.

### STUDY SELECTION

Studies were selected through the following steps:

1. identification of records;
2. removal of duplicates;
3. screening of titles and abstracts;
4. full-text assessment of potentially eligible articles; and
5. application of eligibility criteria to define final sample.

Searches identified 27 records in PubMed, 8 in VHL, 17 in Google Scholar, and 1 in SciELO, for a total of 53 records. After 5 duplicates were removed, 48 studies underwent screening. Of these, 30 were excluded because they did not meet the eligibility criteria, resulting in a final sample of 18 articles.

Figure 1 presents the flow diagram of the study search and selection process, prepared in accordance with the Preferred Reporting Items for Systematic Reviews and Meta-Analyses recommendations.

**Figure 1.**
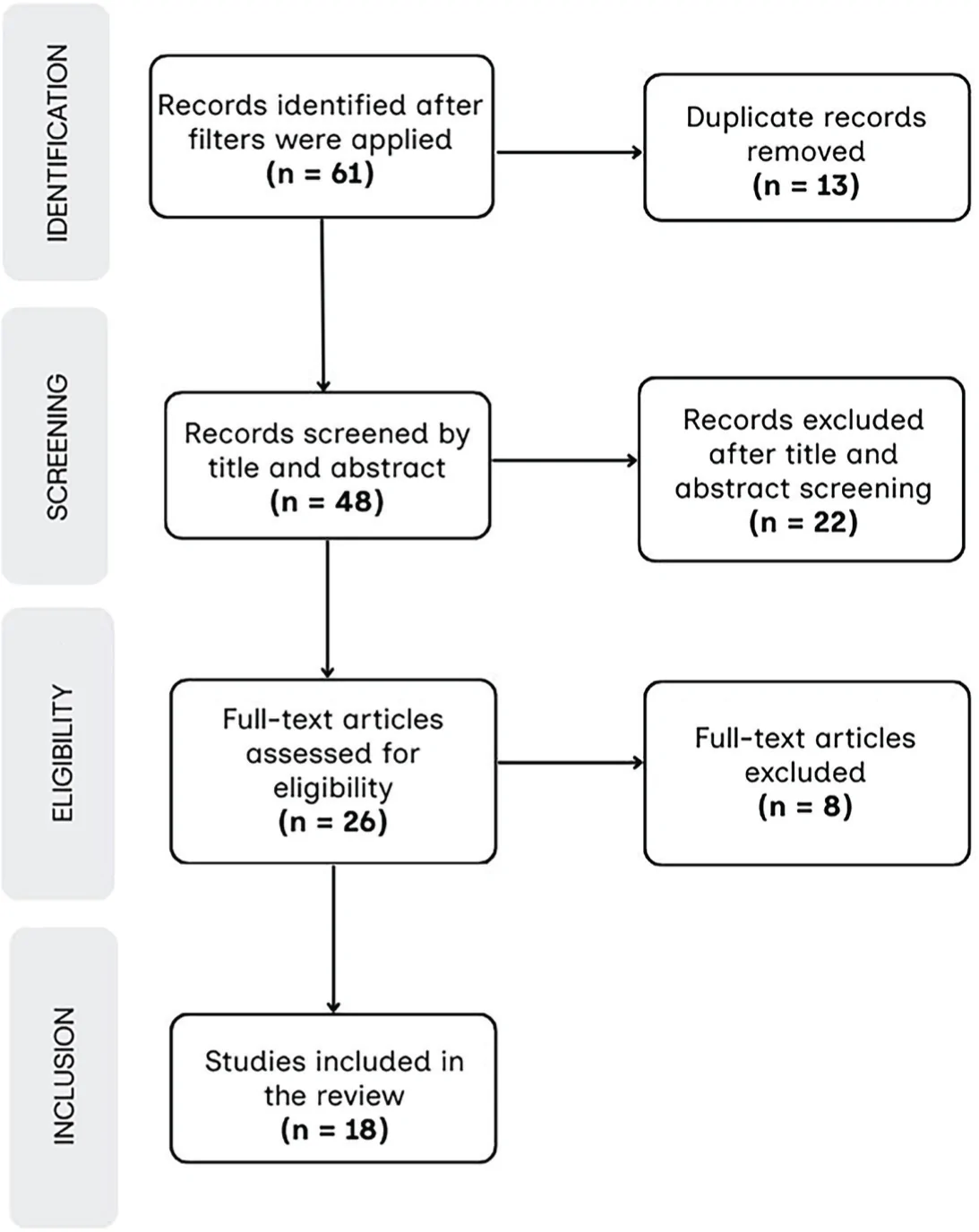
Flow diagram of the study search and selection process in accordance with the Preferred Reporting Items for Systematic Reviews and Meta-Analyses recommendations.

Source: Prepared by the authors (2026).

### DATA EXTRACTION AND ANALYSIS

Data from selected publications were systematically extracted and organized in a structured spreadsheet. Information on title, authorship, year of publication, country/region where the study was conducted, study design, population characteristics, and main outcomes of interest were collected.

Because of methodological heterogeneity of included publications, data were subjected to descriptive analysis and narrative synthesis. Original studies reporting primary data were included in the synthesis of the results, whereas reviews, guidelines, individual case reports, and other publications without primary data were used, when relevant, to provide the theoretical foundation for the Introduction and Discussion.

Table 1 presents the characteristics of selected publications, including authorship, year of publication, study design, population evaluated, and main findings.

**Tabele 1.** Characteristics of selected publications

Authors	Year	Study design	Population or data source	Main findings
Aksoy et al. ^[5]^	2023	Cross-sectional study	Health professionals in Türkiye (n = 98)	Prevalence of sensitization was 9.2% for latex and 17.3% for chemical additives. Most complaints (85.7%) were attributed to irritant dermatitis resulting from excessive hand hygiene rather than allergic mechanisms.
Chiriac et al. ^[11]^	2020	Follow-up study	Health professionals in Romania (n = 245)	Powdered gloves were identified as main triggering factor. Analyses detected cadmium and calcium on inner surface of gloves, possibly contributing to cutaneous toxicity.
Drozdowska et al. ^[12]^	2023	Questionnaire-based study	Employees of a multispecialty hospital in Poland (n = 230)	Nurses and paramedics had greater exposure. More than 10 years of professional experience was associated with a higher level of occupational exposure.
Larese-Filon et al. ^[13]^	2021	Systematic review	Cohort studies and registries from several countries (n = 16)	Incidence of occupational dermatitis was underestimated and significantly higher among trainee nurses than among experienced professionals, a finding attributed to the healthy worker effect.
Gunasegaran et al. ^[2]^	2024	Review	Scientific articles published between 2013 and 2022 (n = 49)	Mean prevalence of hand dermatitis increased from 21% to 37% during pandemic. The use of glove liners and hand hygiene products containing emollients was associated with improvements of 70%-90%.
Hu et al. ^[14]^	2020	Descriptive, quantitative study	Frontline health professionals in Hubei, China (n = 61)	Frequency of reactions associated with latex gloves during COVID-19 outbreak was 88.5%. Predominant symptoms were dryness (55.7%) and pruritus (31.2%).
Ho et al. ^[15]^	2022	Cross-sectional study	Health professionals in Singapore (n = 416)	Prevalence of PPE-associated dermatoses was 73.8%. Continuous glove or mask use for more than 1 hour was associated with a 4.8-fold higher risk of skin lesions.
Jaber & Prasad ^[6]^	2023	Cross-sectional study	Dental professionals in the United Arab Emirates (n = 432)	Latex allergy was identified as the cause of 66.2% of eczema cases. Women reported symptoms more frequently, including skin dryness (19.2% vs. 7.8% among men).
Knežević-Krajina & Knežević ^[16]^	2021	Cross-sectional, pilot study	Health professionals in Croatia (n = 455)	Approximately 50% of participants reported glove-associated skin problems. In 89.8% of cases, symptoms resolved completely during vacations or periods of leave from work.
Loi et al. ^[17]^	2022	Quality improvement project	Health professionals at a tertiary hospital in Singapore (n = 21)	Institutional interventions, such as adopting less irritating products and replacing latex gloves with nitrile gloves, reduced signs and symptoms of dermatitis by approximately 80%.
Ngamchokwathana et al. ^[18]^	2024	Analytical cross-sectional study	Nursing staff in Thailand (n = 481)	Adoption of powder-free gloves with a low protein content (< 50 µg/g) reduced frequency of allergic symptoms from 9.8% to 1.2%.
Pereira & Wilbert ^[9]^	2024	Ecological epidemiological study	SINAN notifications from Brazil between 2018 and 2022	Nursing technicians and surgical center workers accounted for 21.9% of notifications. The hands were the most frequently affected body region, accounting for 63.56% of cases.
Parisi et al. ^[8]^	2021	Review	International research on epidemiological aspects of latex allergy	Latex allergy remains a global problem, with marked inequalities between developed and developing countries in terms of condition management and data availability.
Pourani et al. ^[19]^	2020	Cross-sectional study published as a letter	Health professionals in Iran (n = 390)	Prevalence of contact urticaria during pandemic was 8.2%. Half of cases first manifested during the period of intensive PPE use.
Sakkaravarthi et al. ^[3]^	2022	Cross-sectional study	Health professionals in India (n = 1,088)	Prevalence of allergy was 9.1%. Atopy was associated with a 20.5-fold higher risk, whereas using hand hygiene products more than 10 times per day was associated with a 5.2-fold higher risk.
Tomy et al. ^[4]^	2021	Cross-sectional study	Nurses at a tertiary hospital in India (n = 700)	Prevalence of allergic symptoms was 10.6%. A history of allergy to other rubber materials was associated with a 17-fold higher risk of dermatitis.
Vale et al. ^[7]^	2023	Review	Research on occupational exposure and sensitization	Occupational exposure influences sensitization profiles. The use of synthetic, powder-free gloves was identified as one of main preventive measures.
Henning et al. ^[1]^	2021	Review	Studies involving physicians from different specialties	Physicians, particularly surgeons, were at high risk of dermatitis associated with contact with disinfectants and gloves. Condition was frequently underreported because of professional concerns or self-medication.

Source: Prepared by the authors (2026).

PPE = personal protective equipment; SINAN = Brazilian Notifiable Diseases Information System.

Data from SINAN were analyzed separately and used as a supplementary source to characterize Brazil’s context. Analysis focused on availability and specificity of epidemiological data recorded in the system, particularly regarding the identification of latex as an etiologic agent. Data from technical reports and international surveillance systems were also analyzed as supplementary sources to contextualize availability of epidemiological information on this condition worldwide.

No inferential statistical analyses were performed. Data were analyzed descriptively and synthesized narratively, considering the characteristics and heterogeneity of sources consulted.

### ETHICAL CONSIDERATIONS

Because current study exclusively used publicly available secondary data presented in aggregate form and containing no personally identifiable information, it did not directly involve human participants and was not submitted for review by a research ethics committee, in accordance with Resolution No. 510/2016 of the Brazilian National Health Council. All information was used in accordance with ethical principles applicable to scientific research, with due regard for data integrity and proper acknowledgment of sources consulted.

## RESULTS

### EPIDEMIOLOGICAL SITUATION IN BRAZIL

No Brazilian epidemiological studies were identified that directly estimated prevalence of occupational dermatitis specifically associated with glove use among health professionals.

Although Brazilian literature recognizes occupational latex exposure as a risk factor for developing dermatoses, population-based and multicenter studies capable of estimating the extent of this condition in Brazil remain scarce ^[9]^.

SINAN search was conducted through the DATASUS TABNET module and included records of occupational dermatoses from 2020 to 2025, stratified by etiologic agent. When the “rubber” category was selected to represent latex exposure, no specific reports of occupational dermatitis associated with this agent were retrieved for study period (Table 2).

**Tabele 2.** Number of occupational dermatitis notifications by etiologic agent in Brazil, 2020-2025

Agent	2020	2021	2022	2023	2024	2025
Unknown/blank	99	67	105	133	186	39
Plastic	2	3	—	3	5	1
Solvents	3	8	6	9	16	3
Greases	1	2	1	2	1	—
Cutting oil	2	—	2	3	3	2
Resins	1	2	3	5	3	1
Nickel	2	—	2	6	2	2
Cosmetics	2	—	3	8	6	1
Chromium	9	11	27	49	62	5
Other	6	10	15	15	49	14
Total	127	103	164	233	333	68

Source: TABNET Win32 3.3 — Investigation of occupational dermatoses: notifications recorded in SINAN Net, Brazil.

SINAN = Brazilian Notifiable Diseases Information System.

### INTERNATIONAL DATA

Analysis of international surveillance systems provided context for the burden of occupational dermatoses and their distribution across different sectors and etiologic agents.

Data from occupational surveillance systems, such as THOR in the United Kingdom and THOR-Republic of Ireland, demonstrated that contact dermatitis was the predominant manifestation among work-related skin diseases. This condition accounted for approximately 80%-88% of cases reported in the United Kingdom and 98% of those reported in Ireland ^[20,21]^.

Despite the high proportion of contact dermatitis cases, data from EPIDERM, an epidemiological surveillance system coordinated by the University of Manchester, indicated an overall decline in its incidence. However, this reduction was not uniform across different causative agents, suggesting changes in occupational exposure patterns. The analysis of annual trends by type of contact dermatitis revealed long-term declines of similar magnitude in both allergic cases, which decreased by 5.2% per year between 1996 and 2024, and irritant cases, which decreased by 4.5% per year over the same period. A marked decline in the incidence of allergic cases was observed early in the study period, followed by an increase in ICD, particularly between 2020 and 2024 ^[22]^.

More recent HSE data showed that the health and social care sectors accounted for a substantial proportion of cases, representing approximately 33.8% of those reported between 2020 and 2024 ^[22]^.

Among the occupational exposures identified, rubber materials and PPE, including gloves, were among the etiologic factors reported, although they formed part of a broader set of chemical exposures and occupational activities ^[20,22]^. Available data did not allow the independent contribution of latex to be quantified because exposure categories were often grouped and analyzed together.

### ASSOCIATED FACTORS

#### Most commonly affected occupational groups

Studies demonstrated that occupational contact dermatitis associated with glove use remains a relevant condition among health professionals, particularly those directly involved in patient care activities requiring frequent and prolonged glove use. Nursing professionals were among the most extensively studied groups, with reports of both ICD and latex hypersensitivity ^[3-5]^.

Dentists and other dental professionals were also among the most commonly affected groups, particularly in studies evaluating self-reported occupational skin manifestations resulting from repeated glove use ^[6]^. Studies involving multiple occupational groups also identified cases among laboratory technicians, operating room personnel, and physicians ^[3,5]^.

### RISK FACTORS

Identified risk factors included individual characteristics and conditions related to work environment and occupational activities.

#### Individual factors

Atopy was described as one of the primary individual factors associated with sensitization and the development of latex allergy. In the study by Sakkaravarthi et al. ^[3]^, individuals with a history of atopy had significantly higher odds of sensitization than those without such a history, with an odds ratio of 20.51. The study also identified an association between latex sensitization and hypersensitivity to certain foods, including avocado, banana, kiwi, and nuts.

Atopic conditions, such as allergic rhinitis, asthma, and atopic dermatitis, were also associated with a higher frequency of latex allergy. In the study conducted by Knežević-Krajina & Knežević ^[16]^, approximately half of professionals with glove-associated urticaria had a history of allergic rhinitis, further supporting this association.

The presence of atopic dermatitis or eczema, particularly on the hands, was also associated with impaired skin barrier function. This impairment may facilitate the penetration of latex proteins and chemical additives, such as thiurams and carbamates, thereby promoting immune sensitization and the development of allergic manifestations ^[16]^.

Regarding differences by sex, the studies demonstrated a higher frequency of manifestations related to latex-associated occupational dermatitis among women. In the study by Sakkaravarthi et al. ^[3]^, women accounted for a higher proportion of participants with symptoms than men (72.7% vs. 27.2%) and had a higher rate of positive skin prick tests. Similarly, Jaber & Prasad ^[6]^ observed a higher frequency of self-reported skin symptoms among women, including skin dryness and roughness (19.21% vs. 7.87%) as well as a higher occurrence of hand eczema.

#### Occupational factors

A study involving health professionals in Romania ^[11]^ found an increased risk of skin lesions during the COVID-19 pandemic, associated with intensified hygiene practices and continuous PPE use.

External irritants, such as frequent handwashing and repeated exposure to disinfectants and alcohol-based products, were identified primarily in studies conducted during pandemic ^[14]^. These factors contributed to impaired skin barrier function and may facilitate penetration of allergens, including latex proteins. Although some of these studies did not directly investigate latex sensitization, their findings contributed to understanding the context of skin irritation that may exacerbate subsequent reactions associated with glove use.

Type of glove used in hospital settings was also identified as a relevant factor in developing occupational dermatoses. Gloves with higher latex protein content were associated with a greater risk of sensitization, whereas reducing this protein load proved to be an effective preventive strategy ^[18]^. Furthermore, a pilot study conducted in Croatian hospitals identified an association between using powdered latex gloves and dermatologic changes on hands of health professionals. Powder in these gloves may act as a vehicle for dispersing latex proteins into environment ^[16]^.

ICD was described as the most frequent skin manifestation associated with use of gloves among health professionals. Its primary triggers included increased hand hygiene frequency, continuous use of gloves, and absence of skin-protection measures, such as using emollients. Prolonged use of PPE may also contribute to skin changes, particularly when combined with moisture, friction, and repeated contact with irritants. These conditions impair skin barrier and increase susceptibility to penetration of allergens found in latex or in chemical additives used to manufacture gloves ^[5,12,14,17]^.

### PREVENTIVE MEASURES AND MANAGEMENT STRATEGIES

Latex-associated occupational dermatitis remains a significant challenge among health professionals, with direct effects on quality of life and professional performance ^[4,17]^.

Several preventive strategies have been described to reduce risk of sensitization and dermatitis associated with gloves and other latex-derived materials. Main strategies included replacing latex gloves with latex-free alternatives, such as nitrile or vinyl gloves, in cases of sensitization; using gloves with a low allergenic protein content; and adopting powder-free models ^[18,19]^.

When dermatitis was already established, management strategies included temporarily modifying occupational duties, reducing or eliminating exposure to triggering agent, and, in more severe cases, taking temporary leave from work ^[17]^.

## DISCUSSION

### MULTIFACTORIAL DETERMINANTS

Findings of current review indicate that occupational dermatoses associated with the use of gloves are multifactorial, arising from the interaction between individual predispositions and factors related to work environment and occupational activities ^[2,8]^. Studies analyzed demonstrated that health professionals directly involved in patient care are particularly likely to use gloves frequently and for prolonged periods ^[7]^. In this context, duration and intensity of exposure may influence the risk of developing skin manifestations in hospital settings ^[15]^.

Repeated exposure to irritants, glove-induced occlusion, and frequent hygiene practices contribute to progressive impairment of skin barrier, increasing susceptibility to further irritant damage and allergen penetration ^[13]^. This process may be exacerbated by preexisting skin barrier disorders, such as atopic dermatitis or hand eczema. Furthermore, a history of atopy was associated with a higher risk of sensitization to latex proteins and development of allergic manifestations related to using gloves ^[2,4]^.

Overall, studies consistently indicate that professionals who wear latex gloves for prolonged periods constitute the group at highest risk, regardless of occupational category, because of repeated exposure, prolonged occlusion, and microtrauma to skin barrier. In this context, occurrence of disease depends not only on the presence of an allergen but also on the preexisting integrity of skin, the frequency and intensity of exposure, the quality of gloves used, and the hygiene practices required in workplace.

### INFLUENCE OF GENDER ON OCCUPATIONAL EXPOSURE

Some studies reported a predominance of cases among women, attributing this finding primarily to greater representation of women in occupations involving high levels of workplace exposure, particularly nursing. Evidence indicates that women constitute the majority of the healthcare workforce – estimated at approximately 67% –, which increases their exposure to occupational risk factors ^[2]^.

However, the potential influence of hormonal and immunologic factors on modulation of skin responses to irritants and allergens cannot be ruled out ^[3,5]^.

Thus, the higher occurrence among women appears to be more closely related to their occupational distribution than to an isolated biological characteristic.

### EPIDEMIOLOGICAL GAPS AND UNDERREPORTING

Findings showed that, although recent literature consistently describes risk factors, groups most commonly affected, and prevention strategies, substantial limitations remain in availability of up-to-date epidemiological data, particularly regarding prevalence of latex-associated dermatitis. This gap is especially evident in Brazil, where there is not only an absence of specific records in official databases accessed through DATASUS but also a limited body of scientific literature on the subject. This situation suggests structural limitations in the collection and documentation of such information.

One possible explanation is incomplete or inconsistent completion of etiologic agent field in reporting systems, which reduces ability to identify specific exposures, such as latex exposure. Furthermore, in clinical practice, etiology of dermatitis is not always determined or documented because confirmation of causative agent generally requires specialist evaluation, including patch testing ^[22]^.

These factors contribute to underreporting and possible underestimation of magnitude of problem, despite consistent evidence in literature regarding relevance of latex exposure in occupational settings. Scarcity of national estimates hinders an accurate understanding of the burden of this condition and limits the development of targeted occupational health prevention and surveillance strategies ^[9]^.

In contrast to Brazilian context, international literature provides a larger body of evidence and better epidemiological characterization of occupational dermatoses. Data from international surveillance systems and studies conducted in other countries suggest that work-related dermatoses, particularly contact dermatitis, continue to account for a substantial proportion of occupational diseases and have a significant impact on workers’ health. Nevertheless, even in these settings, lack of detailed stratification by etiologic agent limits specific analysis of role of latex.

### IMPACT OF THE COVID-19 PANDEMIC

Studies consistently document that increased use of gloves during the COVID-19 pandemic raised incidence of hand contact dermatitis ^[2,11,14]^. Recommendations issued by international organizations reinforced need for strict adherence to use of this equipment, intensifying personal protective measures and prolonged use of gloves among health professionals ^[2]^. This situation resulted in an increase in reports of dermatologic conditions associated with using such materials ^[2,11,14]^.

However, analysis of temporal trends reveals a more complex scenario. While some studies reported an increase in cases during the pandemic, data from the United Kingdom showed a marked decline in incidence of allergic cases in late 20th and early 21st centuries. This reduction may be largely attributed to replacement of powdered latex gloves, particularly among health professionals. At the same time, intensification of hand hygiene practices and prolonged use of PPE, a trend that became firmly established between 2020 and 2024, may have contributed to the concurrent increase in ICD ^[22]^.

Accordingly, reduced latex exposure contributed substantially to decline in allergic sensitization. However, this process occurred within a broader transformation of occupational exposures in which irritants assumed a more prominent role, particularly during the COVID-19 pandemic ^[22]^. In this sense, findings do not necessarily indicate a contradiction but rather a shift in profile of occupational dermatoses, possibly related to changes in occupational exposures and characteristics of materials used.

This interpretation should nevertheless be approached with caution, considering limitations of reporting systems, including underreporting, sample variability, and potential effects of pandemic on reporting levels ^[21,22]^. Furthermore, these findings reflect a specific context characterized by well-established regulatory policies, which limits their generalizability to other countries. In settings where such regulations are less stringent or nonexistent, latex exposure may remain high, resulting in a different epidemiological profile ^[3]^.

### INFLUENCE OF REGULATORY POLICIES

Adopting regulatory measures such as manufacturing gloves with low allergen content and reducing or prohibiting use of powdered latex gloves, as well as running public health campaigns, has been associated with a significant decrease in latex sensitization in certain settings. Thus, in countries that have adopted stricter regulations and mandatory warnings, occupational risk tends to be lower because of greater control over the products used ^[8]^.

Conversely, in developing countries, economic constraints that limit access to hypoallergenic alternatives, combined with reliance on educational initiatives as the primary preventive measure, create greater vulnerability to continuous workplace exposure to latex, which may increase the risk of occupational dermatitis. This situation is compounded by underreporting and the scarcity of epidemiological data, which make it difficult to accurately estimate the magnitude of the problem ^[8]^.

Therefore, the differences reported in research may reflect not only temporal variations but also structural inequalities among occupational settings.

### PREVENTION AND MANAGEMENT STRATEGIES

From a pathophysiological perspective, factors such as use of powdered gloves and gloves with a higher protein content contribute to an increased risk of sensitization, whereas concomitant exposure to irritants impairs integrity of skin barrier and promotes development of dermatoses ^[2,7]^.

Findings reinforce the importance of adopting preventive measures in workplace, including switching to hypoallergenic materials and using powder-free gloves with a low protein content ^[2,7]^. These measures are essential to reduce incidence of condition and its effects on quality of life and professional performance.

Educational measures focusing on skin care, such as guidance on skin moisturization, emollient use, and early recognition of manifestations associated with occupational exposure, also play a complementary role in prevention and management ^[2,7]^.

Although these strategies have proven effective in reducing cases, their implementation and adoption may vary according to resource availability and structural conditions of health care services. This finding underscores the need for public policies that expand access to preventive measures and help reduce the burden of occupational dermatoses among health professionals ^[8,17]^.

### LIMITATIONS OF STUDY

Current review has limitations that should be considered when interpreting its findings. Few recent studies published between 2020 and 2025 provided prevalence estimates for occupational dermatitis associated with use of gloves, which limits assessment of current extent of problem.

In Brazil, DATASUS search identified no records associated with rubber, even when broad codes related to dermatoses were used (ICD-10 L20-L30). This result may be attributable to nonmandatory completion of this variable, infrequent documentation of dermatitis etiology, or limitations in how this category is structured within system. In addition, spontaneous replacement of latex gloves with alternative materials and failure of symptomatic workers to seek specialized care reduce the likelihood that episodes of latex-related dermatitis will be officially documented, further limiting availability of data. Consequently, the analysis of data from DATASUS was constrained.

At the international level, analysis was supplemented with data from occupational surveillance systems and regulatory authorities. Although these sources have their own methodological limitations, they provide an indirect estimate of extent of problem in different settings.

Furthermore, heterogeneity among studies in terms of study designs and diagnostic criteria may limit comparability and generalizability of findings.

## CONCLUSIONS

Current integrative review demonstrated that occupational dermatitis associated with latex glove use remains a relevant condition among health professionals, particularly because of its multifactorial nature and its higher occurrence among individuals subjected to frequent and prolonged exposure. Factors such as impaired skin barrier function, atopy, and repeated contact with irritants reinforce interaction between individual susceptibility and occupational environment. Preventive measures, such as use of hypoallergenic materials and appropriate skin care practices, have proven effective in reducing the risk and occurrence of such a condition.

Despite consistency of evidence regarding these factors, availability of recent epidemiological data was substantially limited, particularly in Brazil, and quantitative prevalence estimates for the period from 2020 to 2025 were scarce. Absence of specific records in national databases such as DATASUS contrasts with greater availability of information in international surveillance systems, although these systems also have limitations. In this context, underreporting, gaps in identification of etiologic agents, and lack of standardized reporting compromise accurate measurement of extent of condition.

Finally, expanding scientific research on this topic, strengthening reporting practices, and ensuring accurate documentation of causative agents are essential to determine extent of condition and understand the main settings and exposures involved. These actions may guide more targeted interventions, support development of more effective prevention policies, improve occupational health surveillance, and promote implementation of health management and promotion measures in occupational settings.

## Data Availability

The data supporting the findings of this study are available within the article.
